# A realist evaluation to explain and understand the role of paramedics in primary care

**DOI:** 10.1186/s12916-025-03863-z

**Published:** 2025-01-21

**Authors:** Georgette Eaton, Geoff Wong, Stephanie Tierney, Veronika Williams, Kamal R. Mahtani

**Affiliations:** 1 Nuffield Department of Primary Care Health Sciences, University of Oxford, Oxford, UK; 2 School of Nursing, Nipissing University, North Bay, Canada

**Keywords:** Primary health care, Paramedic, Realist evaluation, Allied health personnel, Extended roles, Additional roles

## Abstract

**Background:**

In response to the unsustainable workload and workforce crises in primary care, paramedics (with their generalist clinical background acquired from ambulance service experience) are increasingly employed in primary care. However, the specific contribution paramedics can offer to the primary care workforce has not been distinctly outlined. We used realist approaches to understand the ways in which paramedics impact (or not) the primary care workforce.

**Methods:**

A realist evaluation was undertaken, consisting of three independent but inter-related research studies:

- In WP1, a mixed-methods cross-sectional survey of paramedics in primary care in the UK was conducted to comprehend the existing practices of paramedics within the NHS.
- WP2 involved an analytic auto-netnography, where online conversations among paramedics in primary care were observed to understand paramedics’ perceptions of their role.
- WP3 utilised focused observations and interviews to delve into the impact of paramedics on the primary care workforce. This comparative study collected data from sixty participants across fifteen sites in the UK, and twelve participants across three sites in a specific region in Canada, where Community Paramedicine is well established.

**Results:**

The culmination of findings from each phase led to the development of a final programme theory, comprising of 50 context-mechanism-outcome configurations (CMOCs) encompassing three conceptual categories: Expectations associated with paramedics in primary care, the transition of paramedics into primary care roles, and the roles and responsibilities of paramedics in primary care.

**Conclusions:**

Our realist evaluation used a mixed-method approach to present empirical evidence of the role of paramedics in primary care. It offers insights into factors relating to their deployment, employment, and how they fit within the wider primary care team. Based on the evidence generated, we produced a series of practice implementation recommendations and highlighted areas for further research.

**Supplementary Information:**

The online version contains supplementary material available at 10.1186/s12916-025-03863-z.

## Background

In the past decade, healthcare policy in the United Kingdom (UK) has increasingly emphasised the integration of non-medical roles to address workforce challenges in primary care [1–6]. These roles include allied health professionals, such as paramedics and physiotherapists, as well as non-clinical roles like social prescribing link workers, and care co-ordinators. Paramedics, as autonomous, degree-educated registered clinicians with extensive experience in ambulance services, are recognised as a versatile addition to primary care [7]. The development of paramedics in the primary care setting is not limited only to the UK. The recognition that paramedicine operates on a health-social care continuum has driven the emergence of community paramedics across Australasia, Canada, and the USA. In these regions, paramedics are integrated into the community to provide primary health care, health promotion, disease management, clinical assessment, and needs-based interventions [8]. Their contributions often play a crucial role in primary care teams, forming key components of local health policy and patient demand plans [9].

However, despite their suitability and policy support for integration, the specific contributions that paramedics can make to the primary care workforce remain unclear. Most research on paramedic roles in UK primary care has overlooked the contextual factors and mechanisms influencing their successful implementation. Understanding these factors is crucial for paramedics to effectively contribute to primary care teams, as current opportunities for their employment in primary care require careful evaluation to benefit patients and align with the National Health Service (NHS) primary care agenda. Evidence must explain and demonstrate how and why their role works, for whom, in what context, and to what extent. Without this understanding, there is a risk of developing a future workforce that is ill-suited to its purpose, lacks clear direction and is unsustainable.

Realist evaluation is a theory-driven research approach that seeks to understand how complex social phenomena (such as the implementation of a new clinical role into an existing primary care workforce) work, focusing on the underlying causal processes that make interventions impactful (or not) in varying contexts [10]. The core of realist evaluation is understanding causation which requires the development and testing (confirmation, refutation or refinement) of the relationship between context, mechanisms, and outcomes. This causal explanation is encapsulated in the context + mechanism = outcome (CMO) configuration (CMOC)—a core analytic and explanatory ‘unit’ in realist research. CMOCs are organised into a programme theory, which provides an overall explanation about context-sensitive causation for a phenomena or intervention. These programme theories are tentative and testable explanations that are a fundamental component of realist evaluation. Realist evaluation is ideally suited to understanding how paramedics work in primary care, as it is already known that their role and impact in primary care are complex. For example, paramedics are trained to different standards and work in a different capacity in different environments [11]. Realist evaluation enables the exploration of these different contexts, how they influence outcomes, and through which mechanisms.

**Fig. 1:**
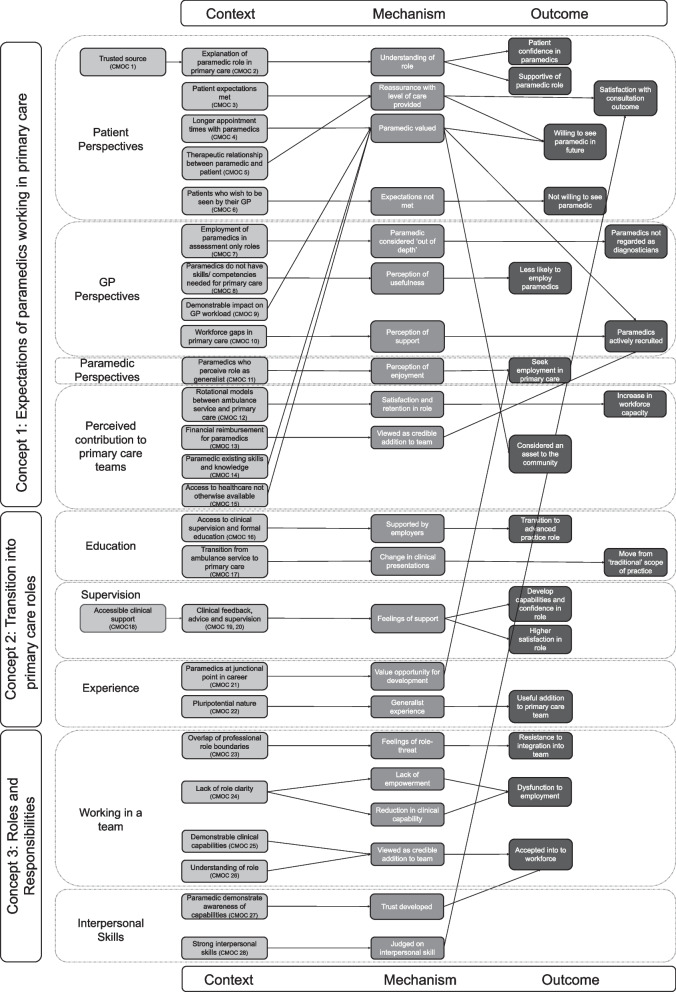
Middle-Range Programme Theory developed from the realist review

Our research sought to answer the question, “What is the role of paramedics working in NHS primary care?” Our research aim was to improve the understanding of the ways in which paramedics impact (or not) the primary care workforce in NHS primary care. This study builds on insights gained from our previous realist review [7], with the goal of refining and expanding our existing programme theory (Fig. 1). This evaluation is reported according to the RAMESES II reporting standards for realist evaluations [12].

## Methods

Primary data were collected from the ‘real world’ NHS practice to further refine the programme theory established during our realist review [7]. Realist evaluation can employ a range of methods to gather relevant data [13, 14]; hence, three distinct, but interrelated, work packages (WP) contributed to this realist evaluation:

- WP1: A cross-sectional survey of paramedics working in primary care [15];
- WP2: An analytic auto-netnography [16];
- WP3: Focused observations and interviews of paramedics, patients they have seen and other professionals they work within primary care [17].

Across these three work packages, data were collected to develop a set of partial programme theories with supporting CMOCs, using formal theory to interpret our findings in light of existing research and knowledge. These partial theories were then integrated to produce a revised, evidence-informed programme theory.

### WP1: a cross-sectional survey of paramedics working in primary care

#### Data collection

A novel, mixed-methods, survey instrument was designed in collaboration with key stakeholders and a Patient-Public Involvement (PPI) group. In brief, the survey gathered data about the demographics of paramedics working in primary care and the extent to which they saw a range of clinical presentations and performed various clinical examination skills within their role. A protocol was published and registered with Open Science Framework (OSF) Registries (10.17605/OSF.IO/YKDA7) and this study was approved by the Central University Research Ethics Committee (MS IDREC Ref: R64129/RE001) at the University of Oxford. The methods and findings have been published elsewhere [15].

#### Recruitment and sampling

A mixture of convenience and snowball sampling was used to distribute the survey to paramedics working in primary care. A total of 341 participants responded from across all four UK nations. This accounted for approximately 33% of the primary care paramedic workforce in England and Wales. However, similar estimates for Northern Ireland and Scotland were unavailable due to the absence of workforce datasets tracking paramedics in primary care roles.

### WP2: an analytic auto-netnography

WP1 identified the need for further investigation regarding: (a) how paramedics are integrated into the primary care workforce to enable the best utilisation of their skills and abilities; (b) whether paramedics maintain their existing professional identity as they move into primary care; and (c) if this is required for them to work in this clinical setting. To address these gaps, further understanding of paramedics’ perspectives of the culture and context of primary care was needed.

#### Data collection

The study designed to address these knowledge gaps was an analytic auto-netnography, situated across three social media platforms where UK paramedics discussed their work in primary care. A protocol was published and registered with OSF Registries (10.17605/OSF.IO/BJQXP) and the study was approved by the Central University Research Ethics Committee (MS IDREC Ref: R77299/RE001) at the University of Oxford.

#### Recruitment and sampling

Over a 3-month period (December 2021–February 2022), Facebook, Reddit and Twitter were searched daily for conversations where the terms featuring ‘paramedics’ and ‘primary care’ were discussed. The primary researcher (GE) observed the conversations, comments and opinions posted within these communities within a reflexive journal, considering them against the context of her own experience.

### WP3: focused observations and interviews

Whilst WP2 contributed to understanding how paramedics perceive their role and work in primary care, gaps in the evidence remained. These included (a) how paramedics can best transition into primary care roles from the ambulance service, (b) the impact of paramedics working in primary care on primary care teams, and (c) the experiences of patients who have a clinical consultation with a paramedic in primary care. This WP addressed these gaps by collecting data from one of the world’s inaugural community paramedic programmes in Canada and considering it alongside data collected previously from the UK [17]. By collecting data from a well-established community paramedic programme, we were able to test and refine the programme theory, ensuring its relevance and applicability across diverse contexts beyond a single geographic area.

#### Data collection

The study design consisted of focused observations and semi-structured (realist) interviews with paramedics working in primary care, patients who had received a consultation with the paramedic, and healthcare professionals and administrative staff working with the paramedic. Data collection took place in the UK and Canada, adopting a comparative approach. The interview guide was developed based on the existing programme theory and in collaboration with the study’s stakeholder group. A protocol was published and registered within the NIHR Central Portfolio Management System (PID16039).

This study was approved to be undertaken in the UK by the Central University Research Ethics Committee at the University of Oxford and the Health Research Authority (22/NW/0097). It was approved to be undertaken within the County of Renfrew Paramedic Service, Ontario, Canada by the Oxford Tropical Research Ethics Committee (OxTREC Reference: 524–22) which was accepted as valid by the County of Renfrew Paramedic Service and Fanshawe College, London Ontario.

#### Recruitment and sampling

Participants were paramedics, general practitioners (family physicians in Canada), patients or carers who had previously had a consultation with a paramedic, and other clinical or administrative members within the primary care team. Each paramedic was considered a ‘case’, around which further data (from other participants) were collected.

In the UK, the sampling framework was purposive, using a maximum variation approach [18] to include individuals who differed in terms of factors relevant to the emerging programme theory, such as length of time as a paramedic, length of time in primary care, job title, and level of education. In Canada, paramedics working within the County of Renfrew Paramedic Service community paramedic programme were convenience-sampled by the Chief Paramedic and Director of Emergency Services.

A sampling framework of 15 cases (paramedics) was considered sufficient to demonstrate the breadth of practice across the UK [19] and to contribute to programme theory development. In Canada, for pragmatic reasons (such as the researcher’s time in the country and eligibility of access), three cases (paramedics) were considered sufficient [20].

## Data analysis

We approached data analysis in two ways. Quantitative data from WP1 was statistically analysed in IBM SPSS Statistics, V.28 (IBM). Qualitative data from WP1, WP2 and WP3 were analysed independently, during the conduct of each, using semantic level, inductive thematic analysis [21] in NVivo V.12. All data were sorted into abstract categories and a realist logic of analysis was then applied to develop contexts, mechanisms, and outcomes within each theme to explain how an outcome was caused by the interaction between the context and mechanism [22]. As CMOCs were developed, they were evaluated against substantive theories to assess their plausibility and analogy with existing knowledge. The CMOCs were sequentially tested using data from each subsequent WP; overlap and duplication were reduced by consolidating them. At each stage, CMOCs were presented to the PPI group and key stakeholder representatives. Discussions with these groups were used to confirm, refute, or refine the CMOCs and to understand how they fit into the overall programme theory.

## Results

**Fig. 2:**
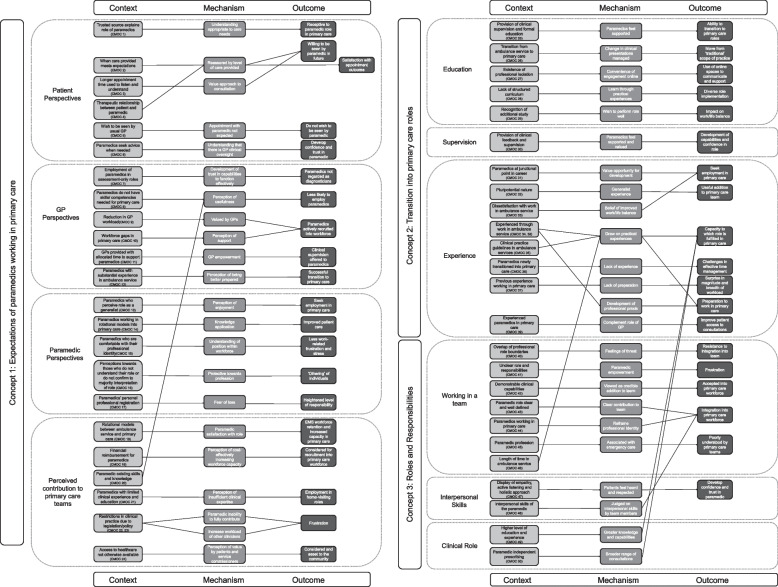
Final programme theory

Following the overall synthesis of findings of the combined data sources within this realist evaluation, the final programme theory comprised 50 CMOCs (Additional file 1) and is outlined visually in Fig. 2. Additional file 2 provides more detail on the development of each CMOC and contains information on the data sources that support the development of each CMOC, derived from each WP. The overall synthesis presented in this paper is a refined programme theory that has developed the three conceptual categories found during the realist review [7] and outlines how, why, to what extent and in what contexts paramedics work in NHS primary care.

### Conceptual category 1: expectations of paramedics working in primary care (CMOCs 1–24)

Patients show receptivity to the role of paramedics in primary care when they receive a clear explanation that these healthcare professionals are integral members of the primary care team and are available for specific consultations. Consequently, when paramedics utilise their extended appointment times to build rapport and connections with patients, individuals express their willingness to have subsequent appointments with paramedics, valuing the additional time during which they feel heard. Patients also show understanding when paramedics need to collaborate with other healthcare providers for their care, recognising the paramedic’s role is within a broader healthcare team, and so patients are amenable to the paramedic seeking advice and input from general practitioners (GPs). However, patients are not receptive to seeing a paramedic when they expect to see a GP for their appointment.

When GPs establish confidence in a paramedic’s competence, they perceive them as a valuable asset to the primary care team and actively seek their inclusion. GPs tend to favour paramedics who have had substantial experience in an ambulance service, as they are seen as better equipped for primary care roles. If GPs do not view paramedics as independent clinicians who can make a diagnosis, they may assign them assessment-only responsibilities. GPs are more inclined to provide clinical guidance to paramedics when they have available time during their workday.

Paramedics are more inclined to pursue careers in primary care when they believe that their education and prior experience in an ambulance service have adequately prepared them for this transition. Additionally, paramedics who engage in rotational roles between ambulance service and primary care find it easier to transfer their skills between these two environments. However, in primary care, paramedics may sense a greater degree of professional responsibility, leading to concerns about their professional registration. Paramedics who are confident in their clinical roles and their contributions to the primary care workforce tend to experience fewer frustrations in this setting. Nonetheless, they are protective of their responsibilities and may distance themselves from individuals they perceive as not aligning with their expectations or understanding of the paramedic role.

In England, employers are more inclined to hire paramedics into their workforce when they receive financial reimbursement from NHS England. Generally, paramedics are actively sought in primary care when their knowledge and experience gained from the ambulance service are deemed valuable for the primary care workforce. Paramedics with limited education and clinical experience may find employment in home-visiting roles, as employers may perceive them to have inadequate clinical expertise for more complex decision-making. Implementing rotational employment models, where paramedics alternate between primary care and ambulance services, has the potential to boost capacity in the primary care workforce while retaining paramedics in ambulance service roles. When paramedics enhance patient access to healthcare, their role is highly appreciated by both primary care teams and patients. However, when legal or policy constraints prevent paramedics from addressing the entire spectrum of conditions encountered in primary care, it leads to frustration for both paramedics and other healthcare professionals, limiting their ability to make meaningful contributions to the primary care team.

### Conceptual category 2: transition into primary care roles (CMOCs 25–39)

Paramedics are motivated to transition to primary care to enhance their clinical skills, leveraging their prior experience in ambulance services as a valuable foundation for work in this setting. They find the transition smoother and more supportive when they have access to clinical supervision and structured educational opportunities. In cases where formal education is lacking, paramedics acquire knowledge through practical experience in primary care. Managing their time efficiently in a primary care setting can pose challenges. Clinical supervision plays a crucial role in honing their capabilities and fostering confidence in their new roles. As they transition, paramedics expand their scope of practice, adapting to different clinical conditions from those encountered in the ambulance service; this can lead to variations in the paramedic role due to a lack of standardisation. Rather than being a problem, this versatility allows the paramedic role to be effectively utilised to address specific gaps in the healthcare system, including attending to a range of patient presentations via telephone triage, urgent appointments, routine consultations, long-term condition reviews, and home visiting. In essence, experienced paramedics complement the work of GPs and enhance workforce capacity by improving patient access to consultations.

### Conceptual category 3: roles and responsibilities (CMOCs 40–50)

For paramedics’ effective integration into the primary care team, it is crucial to have well-defined roles and responsibilities; when this is lacking, there may be blurring of professional boundaries causing tension with other staff and impeding effective team integration and working. When their capabilities and contributions are clearly defined and they possess the requisite knowledge and experience to support their roles, paramedics are recognised as a valuable addition to the workforce. However, the role and potential contributions of paramedics are often poorly understood by the primary care team, so paramedics need to redefine their professional identities for successful integration. Strong communication and interpersonal skills are essential for achieving this integration, and they also help patients develop trust in paramedics. In summary, paramedics with higher levels of education and clinical experience, including independent prescribing capability, can more effectively assume an expanded clinical role in primary care, allowing them to conduct a wider range of consultations.

## Integration with substantive theory

During the development of the initial programme theory in the realist review, we critically examined established substantive theories related to professional role boundaries, professional identity, and transition (liminality) that supported conceptual categories within it. During the refinement of the programme theory, as empirical data were collected in the realist evaluation, these theories continued to be relevant.

Professional identity remains a relevant substantive theory that has helped us to better understand parts of our programme theory. However, there has been a shift in the sociological theories that we use. Whereas exploration of this conceptual category was initially influenced by the work of Freidson [23], and the professionalism of the paramedic, the knowledge generated in WP2 and WP3 indicated that paramedics are proactively reshaping their professional identity while working in primary care so that they may more effectively integrate into this workforce. Goffman’s theory of ‘frame alignment’ [24] adds further clarity, describing how individuals adjust their cognitive frames to align with others during social interactions to achieve integration within a new context. Therefore, the integration of paramedics in primary care hinges on their level of comfort with their professional role within this workspace, and their capacity to align their clinical practice with the requirements of their role. As paramedics move from a *blue-collar* ambulance-based role into a specialised workspace, such as primary care, this brings into tension the expectations paramedics bring within them about their role and how that role should be practised [25]. This demonstrates an unexpected paradox, where paramedics in primary care viewed their identity as significantly different from the work of their profession in the ambulance service, but similar enough to retain the *vestigia* of paramedicine.

The realist review [7] found that transitioning into primary care can be seen as a threshold concept (a core principle that changes the way a person understands a field, or new setting such as primary care) signifying fundamental changes in the practice of the discipline. Understanding these changes is essential for clinicians to progress effectively [26]. Paramedics transitioning into primary care can occupy a state of liminality (a transitional period where individuals are betwixt and between their previous state and the new one), during which there is only a partial understanding of how they can ‘work’ in their new role [27]. Data outlined above develops an understanding of this further, highlighting that as paramedics move through this liminal state (where they are neither in their old role in ambulance services nor fully integrated into primary care), they navigate a sense of ambiguity by seeking clinical support, particularly from GPs. This finding resonates with Bridges’ transition theory [28]; it highlights the importance of support, learning, and adaptation during the process of transition.

Related to this is the importance paramedics place on the provision of formal education (such as taught master’s degrees) to facilitate their integration into primary care, a finding widespread in WP3. This distinctly Vygotskian perspective indicates that these paramedics see education as a route to successful integration into primary care; the more they know, the more patients they can see, and the more effective they are in primary care [29]. WP2 identified that in the absence of formal education, paramedics develop their own communities of practice using online social spaces, which appear to play a crucial role in anticipatory socialisation [30] for paramedics to then enter the primary care workspace. This demonstrates that support-seeking behaviour to aid transition does not need to be limited to a formal supervision structure from physician to paramedic but can also be provided amongst peers who navigate together the challenges and differences in their new environment [31].

Data from this evaluation highlighted the importance of professional role boundaries and the impact of paramedics working in primary care teams. Interviews with GPs and other clinical staff revealed no concerns about role substitution or conflict, reinforcing findings from our realist review [7]. Paramedics were seen as complementary to the GP role, a view echoed by nurses and clinical pharmacists who felt that paramedics fit well within their teams without duplicating or infringing upon their clinical roles. Conceptually, this underscores the role of team process theories in navigating professional role boundaries in primary care, where team effectiveness is achieved when team processes align with task demands [32]. For example, paramedics can help meet increasing patient demand by assessing patients and providing treatment or referrals. However, if paramedics do not work autonomously and, therefore, increase the GP’s workload, team effectiveness suffers. This supports recent research on primary care workforce composition which found that GP satisfaction does not increase with skill mix changes [33].

## Discussion

Our realist review [7] indicated that paramedics, by virtue of their experience in the ambulance service, are generalist clinicians. Their generalist background affords them pluripotency, which can be advantageous in primary care, provided they receive appropriate clinical supervision and support to facilitate their transition into this setting.

Summarising the final programme theory from this realist evaluation, paramedics work in primary care to enhance patient access, manage specific conditions, and complement the role of the GP. They typically handle non-urgent cases, leveraging their generalist background to meet their practice’s specific needs, which can vary widely. For instance, some paramedics work in minor injury clinics, others conduct home visits, and some follow a routine appointment schedule, like GPs. This flexibility allows GPs to focus on complex cases while ensuring patients receive timely care from an autonomous healthcare professional.

While paramedics possess a broad range of clinical skills, this evaluation demonstrates further education is required in the management of specific conditions, moving beyond the recognition and diagnosis that was the focus of their ambulance service work. The significance of clinical experience gained in the ambulance service, and its relevance in terms of clinical competence in primary care, was underscored in every phase of the realist evaluation. These clinical experiences contributed to the integration and effectiveness of paramedics as a professional group within the primary care team.

The importance of interpersonal skills was also highlighted in the realist review and found throughout each WP of this evaluation. Patients, in particular, reacted positively to paramedics in primary care due to their ability to establish rapport, in addition to their clinical role and providing effective care.

Paramedics have the potential to improve healthcare access, particularly in rural areas or regions with challenges in recruiting GPs and other clinicians. In these situations, paramedics do not replace the role of GPs or other clinicians but can provide timely access to care for patients who may otherwise face extended waiting times to be seen.

## Strengths, limitations and future directions

This realist evaluation was enriched by its utilisation of a diverse array of methods and data sources, gathering empirical data from various perspectives, encompassing statistical data, perceptions, observed experiences, and interviews. Data collection spanned three years, with each WP completed consecutively, allowing for the capture of role developments over time. However, it is important to note that these findings provide momentary, cross-sectional glimpses of the paramedics’ position in primary care, and thus may quickly become obsolete as the profession evolves to address the changing needs of primary care.

The realist approach introduces a potential risk of selective bias in the substantive theory used to develop the programme theory. To mitigate this risk, the research team embraced a participatory approach by involving representatives from various stakeholder organisations as well as a patient and public participant group in a collaborative effort to develop the CMOCs. These contributions enabled the refinement of the evolving programme theory and examination of the implications of the findings. However, it is essential to remember that the programme theory proposed in this evaluation is an approximation of reality and should be subject to testing and refinement in future studies.

Whilst the programme theory offers a collection of underlying explanations regarding how paramedics work in primary care, a gap within this programme theory concerns the operationalisation of the effectiveness of paramedics working in primary care, for example measurement of clinical outcomes, efficiency measures, safety measures or cost-effectiveness. Future research should look to use a combination of these measures to obtain an understanding of clinical effectiveness, which may also result in more efficient use of paramedics as a resource within the primary care team.

## Comparison with existing literature

Despite being a key policy area, there remains minimal additional research evidence regarding paramedics in UK primary care. However, research focusing on the barriers and facilitators of the transition of paramedics to primary care supports the recommendations identified in this evaluation, namely addressing gaps in education and role misunderstanding [34]. A recent rapid realist synthesis [35] highlights similar findings to our previous realist review [7] and the programme theory developed from this realist evaluation, namely the variation in paramedic roles within primary care across the UK, general understanding of the paramedic role within primary care teams, and the importance of clinical supervision to aid integration of paramedics into primary care. In addition, the findings of our realist evaluation, and resulting programme theory, are comparable to international literature regarding community paramedics [9, 36], where the importance of further education to support integration into primary care and enhance the clinical contribution paramedics can make in this setting.

Recent UK policy recommendations support these findings but also highlight that rotational models of working—where paramedics are employed by the ambulance service and rotate into primary care—do not achieve true integration of paramedics into primary care settings [37]. Our programme theory found no issues relating to integration, but did find that paramedics who engage in rotational roles between ambulance service and primary care find it easier to transfer their skills bidirectionally between these two environments (CMOC 14) and that such portfolio models increase paramedic staff satisfaction in their role (CMOC 18). We also found that the implementation of rotational employment models has the potential to boost capacity in the primary care workforce while retaining paramedics in ambulance service roles, which correlates to previous findings [38]. Given the rise in portfolio working among paramedics, especially advanced paramedics within UK ambulance services, this area demands closer scrutiny to ensure its effectiveness and accuracy.

## Implications for practice and policy

Based on the findings, there are four key recommendations regarding how paramedics work in primary care:

1. A clear strategy for communication of the paramedic’s role in primary care

The importance of informing patients regarding the presence of paramedics in their primary care practice, as well as the specific situations in which they might interact with a paramedic, was underscored as essential for fostering patient comprehension of the role and establishing clear expectations about their appointments (CMOCs 1–6). However, it was also demonstrated that the paramedic role is not well understood by primary care teams (CMOC 45), and this can impact the integration and work of the paramedic in primary care (CMOCs 7, 8, 10, 20, 40–46).

The importance of a clear communication strategy regarding the employment of paramedics in primary care, which is accessible to patients and understood by clinical and non-clinical members of the team, is important in supporting this integration. Such a strategy may include the following:

- Introduction of the paramedic to the patients registered within the practice and their role within primary care (CMOCs 1, 2, 5, 49).
- Outline of their educational background and experience (CMOCs 34, 46, 49).
- Clear expectations about when patients may see a paramedic in the course of their care (CMOCs 1, 6, 39, 40).

2. Developing a comprehensive curriculum framework for paramedics in primary care

The development of a comprehensive nationwide curriculum framework that delineates the fundamental competencies for paramedics operating in primary care settings would facilitate the establishment of a baseline standard for the profession within primary care, whilst also ensuring that the paramedic role in primary care can be adapted to meet the needs of the community it serves, as informed by the evidence generated as part of this realist evaluation (CMOCs 26–29, 50).

3. The need for an effective transition support structure

In order for paramedics to effectively complement the GP role in primary care teams, they need support to transition into working in primary care and successfully integrate into these teams. Such a transition firstly requires recruiting the right paramedic for the primary care team. In particular, paramedics’ capabilities (including their level of education and experience) are important if they are to effectively complement the GP role (CMOCs 9, 10, 18, 24, 50). In addition, their interpersonal skills are crucial in facilitating the integration of the individual within the primary care team and for patients’ acceptance (CMOCs 47 and 48).

**Fig. 3:**
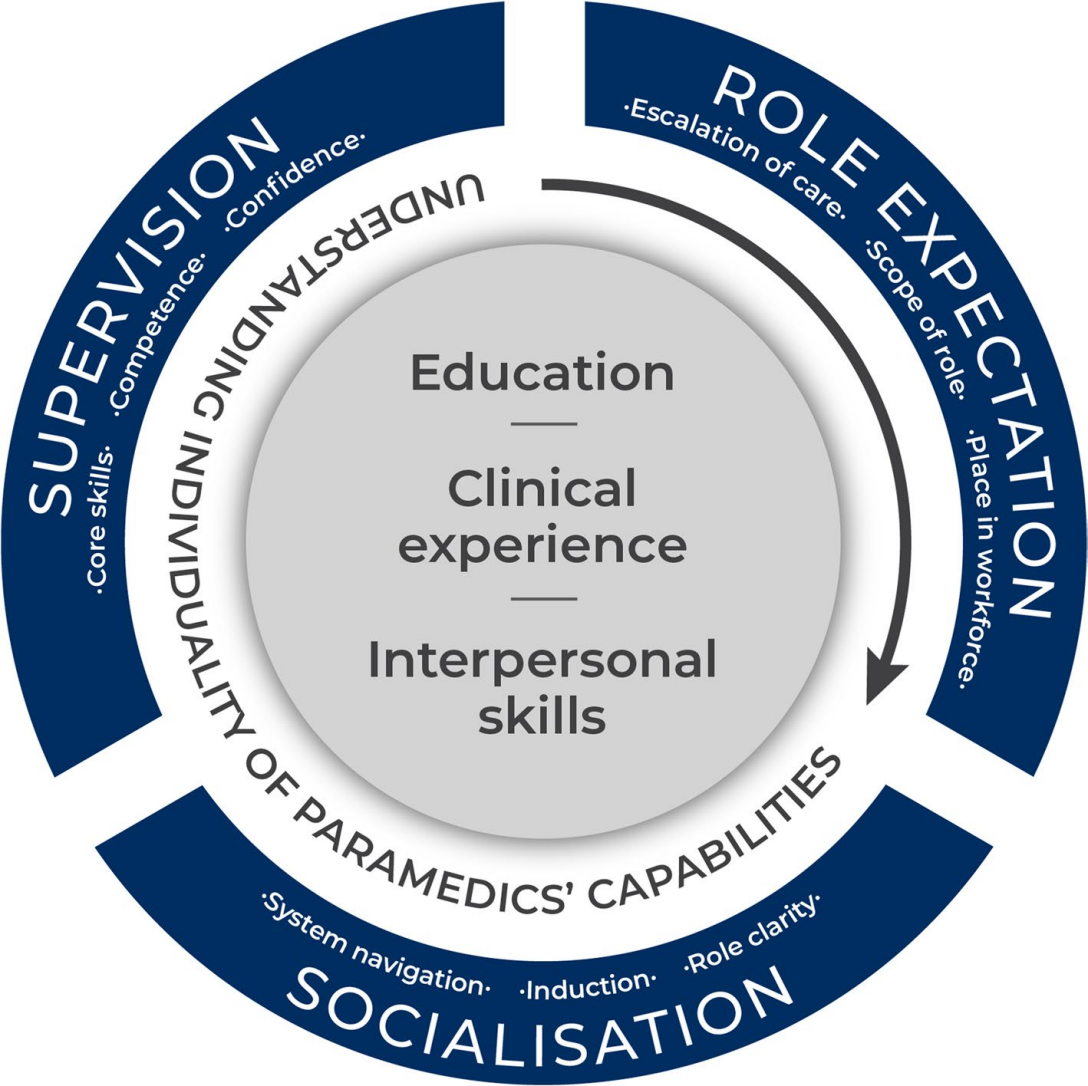
A model to support the transition of paramedics to primary care

Through understanding the individuality of these capabilities, specific support structures for the paramedic to transition effectively into primary care can be adopted. These support structures should revolve around socialisation into the primary care team (CMOCs 7, 8, 12, 13–15, 21), provision of supervision for the paramedic (CMOCs 11, 25, 30–39), and clarity regarding role expectations (CMOCs 40–43). A model to support the transition of paramedics to primary care is recommended in Fig. 3.

4. Changes to legislation and policy

Limitations in legislation (particularly regarding restrictions around the prescription of Schedule 2 controlled medicines or policy supporting the provision of a Statement of Fitness for Work) have an impact on the ability of paramedics to contribute in their fullest capacity to primary care teams (CMOCs 22 and 23). These necessary changes to legislation and policy would support paramedics to provide a meaningful contribution within primary care teams.

These recommendations are further detailed in an online toolkit, available at www.paramedicsinprimarycare.info, which we have developed to provide valuable information for patients, paramedics, primary care staff, and employers [39].

## Conclusions

The final programme theory demonstrates that when deployed appropriately, paramedics in primary care enhance patient access by complementing the role of GPs. However, the absence of formal education in their role requires paramedics to depend on their prior experience in the ambulance service to make contributions to the primary care team. While clinical acumen gained through experience is essential, it should not be the sole foundation for paramedics’ preparation to work in primary care. Relying primarily on experience can lead to significant variations in their contributions, making it challenging to standardise the role of paramedics across the primary care workforce in the UK. Optimal implementation of paramedics in primary care requires a comprehensive approach encompassing a communications strategy for clarifying their role, a curriculum framework defining their necessary capabilities (whilst also promoting versatility in role application), a support structure for a smooth transition into the primary care workforce, and legislative adjustments to enable paramedics to make their fullest contribution to primary care.

## Supplementary Information

Additional file 1. Final Programme Theory.

Additional file 2. Overview of data sources from realist review and illustrative examples of data that have contributed to CMOC development.

## Data Availability

The datasets used and/or analysed during the current study are available from the corresponding author on reasonable request. Data is provided within the manuscript or supplementary information files.

## Abbreviations

CMO: Context + mechanism = outcome
CMOC: Context-mechanism-outcome configurations
GP: General Practitioner
NHS: National Health Service
NIHR: National Institute of Health and Care Research
OSF: Open Science Framework
PPI: Patient-Public Involvement
RAMESES: Realist And Meta-narrative Evidence Syntheses: Evolving Standards
UK: United Kingdom
WP: Work package
