# Supplementary material for: A realist evaluation to explain and understand the role of paramedics in primary care
Source: BMC Med. 2025 Jan 21;23:30. doi: 10.1186/s12916-025-03863-z (PMC11753038; doi:10.1186/s12916-025-03863-z)
Supplement: Supplementary file 1 — Additional file 1. Final Programme Theory. [file 12916_2025_3863_MOESM1_ESM.docx]

**Additional File 1: Final programme theory**

**Concept 1: Expectations of paramedics working in primary care**

***Patient Perspectives***

**CMOC 1**: When a trusted source explains the role of a paramedic in primary care (C), patients understand how the role may be appropriate to their care needs (M), and are receptive to the introduction of paramedics in the primary care workforce (O)

**CMOC 2**: When the care provided by paramedics meets patients’ expectations (C), they will be more willing to be seen by a paramedic in the future (O) and more satisfied with the appointment outcome (O) because they are reassured with the level of care provided (M)

**CMOC 3**: When a paramedic uses their longer appointment time to listen and understand a patient’s problem (C), patients are more willing to see them again (O), because they value this approach (M)

**CMOC 4**: When paramedics have a therapeutic relationship with the patients they see (C), these patients are reassured with the level of care provided (M) so are more willing to be seen by a paramedic in the future (O) and are more satisfied with the appointment outcome (O)

**CMOC 5**: When patients want to be seen by their usual GP (C), they do not wish to be seen by a paramedic (O) as this is not what they expect (M)

**CMOC 6**: Patients develop confidence and trust in paramedics (O) when paramedics need to seek advice about their care if needed (C), because they understand there is clinical oversight from a GP (M)

***GP Perspectives***

**CMOC 7**: When primary care providers do not regard paramedics as diagnosticians (O) they employ them in assessment-only roles (C) until they develop trust in the paramedic’s capabilities to function effectively in primary care (M)

**CMOC 8**: When the employer does not consider the paramedic to have the skills and competencies relevant for their needs (C) employers may be less likely to employ paramedics (O) because they do not consider them to be useful (M)

**CMOC 9**: When paramedics can demonstrate that they can help to reduce the workload of GPs (C), because this is valued by GPs (M), paramedics continue to be actively recruited into primary care (O)

**CMOC 10**: Paramedics are actively recruited into primary care (O) when gaps within the workforce exist (C) because they are perceived to be able to support general practice (M)

**CMOC 11**: When GPs are provided with allocated during their working day to provide support to paramedics in primary care roles (C), they are more likely to offer clinical supervision (O) because they are empowered to do so (M)

**CMOC 12**: Paramedics with substantial experience in the ambulance service (C) are perceived to be better prepared by GPs (M) to make a successful transition into the primary care workforce (O)

***Paramedic Perspectives***

**CMOC 13**: Paramedics who perceive their role as a generalist (C), will look for opportunities for employment in primary care (O) because they believe they can enjoy and work in that environment (M)

**CMOC 14**: Paramedics working in rotational roles between emergency medical services and primary care (C) feel able to apply knowledge learnt in one area to another (M) to provide improved patient care (O)

**CMOC 15**: Paramedics who are comfortable with their professional identity (C) experience less work-related frustration and stress in primary care (O) because they understand their position within the workforce (M)

**CMOC 16**: Paramedics working in primary care perceive colleagues as lacking a comprehensive grasp of their duties, or those who do not conform to the majority's interpretation of the role (C) they tend to "other" those individuals (O) because they are protective toward their profession (M)

**CMOC 17**: Fear of loss of personal professional registration (M) remains important for paramedics in primary care (C) who consider a heightened level of professional responsibility in comparison to their roles in the ambulance service (O)

***Contribution to Primary Care Teams***

**CMOC 18**: When the use of rotational models for paramedics enables them to work and transfer their skills between the ambulance service and primary care (C) the workforce needs of both organisations are more likely to be met (such as EMS workforce retention and increased workforce capacity in primary care) (O) because paramedic staff satisfaction is higher (M).

**CMOC 19**: Paramedics are considered for recruitment into the primary care workforce in England (O) when employers perceive that they are a cost-effective way of increasing the primary care team capacity (M) due to the availability of adequate financial reimbursement for their roles (C).

**CMOC 20**: When the existing skills and knowledge of paramedics are perceived by commissioners and stakeholders to correlate well into primary care (C) paramedics are actively recruited into primary care (O) because what they can offer is considered useful (M)

**CMOC 21**: Paramedics with limited clinical experience and education (C) are usually employed in home visiting roles within primary care (O) because their employers judge they have insufficient clinical expertise for more challenging clinical decisions (M)

**CMOC 22**: When paramedics are prevented by legislation or policy from seeing the full range of conditions that present to primary care (C), they feel frustrated in their role (O), because they feel they cannot contribute in the fullest way (M)

**CMOC 23**: When paramedics are prevented by legislation or policy from seeing the full range of conditions that present to primary care (C), other clinicians in the primary care workforce are frustrated (O), because this increases their workload (M)

**CMOC 24**: When paramedics provide access to healthcare which otherwise would not be available (C) they are considered a community asset (O) because what they do is highly valued by patients and service commissioners and providers (M)

**Concept 2: Transition into primary care roles**

***Education***

**CMOC 25**: When employers provide clinical supervision (C) and access to formal education to paramedics (C) they are better able to transition into primary care roles (O) because they feel supported (M)

**CMOC 26**: As paramedics transition from emergency medical services (EMS) to primary care roles (C), they move away from their traditional scope of practice (O) because of the change in clinical conditions they have to manage (M)

**CMOC 27:** When professional isolation exists for paramedics in primary care (C), paramedics tend to use online social spaces to exchange insights regarding their role (O) and to support each other (O) because it is a convenient way to engage with others (M)

**CMOC 28:** Lack of a structured curriculum to support primary care education (C) means that paramedics learn through practical experience of working in primary care (M) leading to diverse approaches to role implementation (O)

**CMOC 29**: When paramedics recognise that working in primary care requires additional study (C) they will often undertake this in their own time (which has an impact on their work/life balance) (O) because they want to do their job well (M)

***Supervision***

**CMOC 30**: When paramedics are provided with clinical feedback and supervision in general practice (C), because they feel supported and valued (M) they will develop their capabilities and confidence within their role (O)

***Experience***

**CMOC 31**: Paramedics at a junctional point within their career (C) value the opportunity to develop themselves (M) and so look for opportunities for employment in primary care (O)

**CMOC 32**: Paramedics are pluripotent (C). Because of the breadth of issues with which they can deal (M) paramedics are considered a useful addition to the primary care team (O)

**CMOC 33**: Paramedics who are dissatisfied with their work in the ambulance service (C) will look for opportunities for employment in primary care (O) because they believe this will afford them a better work/life balance(M)

**CMOC 34:** Paramedics who are experienced by virtue of their work in the ambulance service (C) fulfil an increased clinical role in primary care (O) because they have practical experience to draw upon (M)

**CMOC 35**: When paramedics are not allowed to see some patient groups due to the clinical guidelines that exist in their work in ambulance services (C) they are prevented from gaining experience in the whole range of conditions that present to primary care (O) because they don't have these relevant opportunities to learn from (M)

**CMOC 36:** When paramedics have newly transitioned into working in primary care settings (C) effective time management poses a challenge for them (O) because they lack the experience needed (M)

**CMOC 37**: When paramedics have no previous experience of working in primary care (C), they are surprised about the magnitude and type of work they are required to do (O) owing to an absence of adequate preparation for such responsibilities (M)

**CMOC 38**: Paramedics consider that their experiences in the ambulance service (C) are important in preparing them to work effectively in primary care (O) as working in the emergency ambulance setting has enabled them to develop their professional praxis (M)

**CMOC 39**: Experienced paramedics in primary care (C) complement the role of the GP (M) and are able to increase workforce capacity (O) by improving patient access to consultations (O)

**Concept 3: Role and Responsibilities**

***Working in a Team***

**CMOC 40**: When the professional role boundaries of paramedics overlap with existing health care professionals in General Practice (C) there may be resistance of the paramedic role and responsibilities by these other health care professionals (O), because they feel threatened (M)

**CMOC 41**: Paramedics experience frustrations in their role (O) when their role and responsibilities are unclear (C). When this occurs, paramedics are less likely to be empowered (M)

**CMOC 42**: When the paramedics capabilities have been demonstrated (C) they are viewed by practice staff as a credible addition to the team (M) and are accepted into the practice workforce (O)

**CMOC 43**: When the role of paramedics is clear and well defined (C) the paramedic integrates well in the primary care workforce (O), because their contribution is clear (M)

**CMOC 44**: Working in primary care (C) requires paramedics to reframe their professional identity (M) in order to integrate into the workforce (O)

**CMOC 45**: The paramedic profession (C) is poorly understood by primary care teams (O), who associate it with the provision of emergency care in an ambulance only (M)

**CMOC 46**: Primary care teams consider that length of experience in the ambulance service (C) is important in preparing paramedics to work effectively in primary care (O) because they have a broad range of practical experience to draw upon (M)

***Interpersonal skills***

**CMOC 47**: Where paramedics display empathy, active listening and a holistic approach to the patient (C) the patient develops trust and confidence in the paramedic (O) because they feel heard and respected (M)

**CMOC 48**: Successful integration of paramedics into primary care teams (O) is attributed to the interpersonal skills and enthusiasm of the paramedic (C) because primary care team members use these to judge the type of person they work alongside (M)

***Clinical role***

**CMOC 49**: Paramedics who have a higher level of education and clinical experiences (C) fulfil an increased clinical role in primary care (O) because they have greater knowledge and capabilities (M)

**CMOC 50**: Paramedics who can independently prescribe medicines (C) fulfil an increased clinical role in primary care (O) because they are able to complete a broader range of consultations (M)
