# Supplementary material for: A realist evaluation to explain and understand the role of paramedics in primary care
Source: BMC Med. 2025 Jan 21;23:30. doi: 10.1186/s12916-025-03863-z (PMC11753038; doi:10.1186/s12916-025-03863-z)
Supplement: Supplementary file 2 — Additional file 2. Overview of data sources from realist review and illustrative examples of data that have contributed to CMOC development. [file 12916_2025_3863_MOESM2_ESM.docx]

| **Additional File 2: Overview of data sources from realist review and illustrative examples of data that have contributed to CMOC development**  **Concept 1: Expectations of paramedics working in primary care** | | | | | |
| --- | --- | --- | --- | --- | --- |
| **Patient Perspectives** | | | | | |
| **Initial CMOC** | **Realist Review** | **Realist Evaluation** | | | **Final CMOC** |
|  |  | **Phase I** | **Phase II** | **Phase III** |  |
| Patients want to know what paramedics do in their General Practice. When the paramedic role in General Practice is clear to patients (C) they understand how the role is appropriate in relation to their care needs (M), and so have increased confidence when they are treated by paramedics (O) | (Dainty et al., 2018; Martin et al., 2016; O’Meara et al., 2016) | “*Some patients do not understand my role/skill set and just want a GP*” (RID 143; Advanced Paramedic), |  | *“having a heads-up that they've got paramedics working there would be good”* (UK804: Patient)  *“I suppose generally we’re very happy with our GP surgery. So yeah… I would be quite trusting of them anyway and whoever they sent”* (UK404: Patient carer) | CMOC 1: When a trusted source explains the role of a paramedic in primary care (C), patients understand how the role may be appropriate to their care needs (M), and are receptive to the introduction of paramedics in the primary care workforce (O) |
| When a trusted source explains the role and value of seeing a paramedic (C), patients understand how the role is appropriate in relation to their care needs (M), and so are supportive of the introduction of these new roles (O) and have increased confidence when they are treated by paramedics (O) |  |  |  |  |  |
| When the care provided by paramedics meets patients’ expectations (C), they will be more willing to be seen by a paramedic in the future (O) and more satisfied with the appointment outcome (O) because they are reassured with the level of care provided (M) | (Brydges et al., 2016; Dalgarno, 2016; Martin et al., 2016; Proctor, 2019) |  |  | *“…when you walk away, have you been dealt with properly or are you not very happy? You know, that's the main thing. And ((name)) certainly consoled me, you know. Put my mind at rest anyway.”* (UK1004: Patient) | CMOC 2: When the care provided by paramedics meets patients’ expectations (C), they will be more willing to be seen by a paramedic in the future (O) and more satisfied with the appointment outcome (O) because they are reassured with the level of care provided (M) |
| When a paramedic uses their longer appointment time to listen and understand a patient’s problem (C), patients are more willing to see them again (O), because they value this approach (M) | (Brydges et al., 2016; Martin et al., 2016; O’Meara et al., 2016; Sibley et al., 2018). |  |  | *“…if they’re like he is and can give you that five minutes extra time and can answer your questions without constantly looking at the screen, then yes I would be more than happy to go to any paramedic.”* (Uk1503: Patient). | CMOC 3: When a paramedic uses their longer appointment time to listen and understand a patient’s problem (C), patients are more willing to see them again (O), because they value this approach (M) |
| When paramedics have a therapeutic relationship with the patients they see (C), these patients are reassured with the level of care provided (M) so are more willing to be seen by a paramedic in the future (O) and are more satisfied with the appointment outcome (O) | (K. Abrashkin et al., 2018; Dalgarno, 2016; Proctor, 2019; Rasku et al., 2021) |  |  | *“I have no qualms about seeing him again, in fact I wanted to see him again today because it was my second appointment and I felt it was good to stay with the same person… If he was going to be my GP for the rest of my life I’d be really happy…”* (UK1402: Patient). | CMOC 4: When paramedics have a therapeutic relationship with the patients they see (C), these patients are reassured with the level of care provided (M) so are more willing to be seen by a paramedic in the future (O) and are more satisfied with the appointment outcome (O) |
| When patients want to be seen by their usual GP (C), they do not wish to be seen by a paramedic (O) as this is not what they expect (M) | (Halter et al., 2007; Proctor, 2019; RSM UK Group, 2017) | Ten paramedics (3% of participants) who responded to the survey outlined sentiments such as “*patients still question why they are being seen by a paramedic*” (RID 76; Advanced Paramedic Practitioner) and “s*ome are only reassured once they see a doctor*” (RID 270; Primary Care Practitioner). |  |  | CMOC 5: When patients want to be seen by their usual GP (C), they do not wish to be seen by a paramedic (O) as this is not what they expect (M) |
| Patients develop confidence and trust in paramedics (O) when paramedics need to seek advice about their care if needed (C), because they understand there is clinical oversight from a GP (M) |  |  |  | *“Interviewer: And you don't mind that she has to go to the GP and chat before she might be able to give you a treatment?*  *Participant: Not at all. Not at all, no because at least you've got to talk to somebody who knows what they're talking about you know.”*  (UK104: Patient). | CMOC 6: Patients develop confidence and trust in paramedics (O) when paramedics need to seek advice about their care if needed (C), because they understand there is clinical oversight from a GP (M) |

| **GP Perspectives** | | | | | |
| --- | --- | --- | --- | --- | --- |
| **Initial CMOC** | **Realist Review** | **Realist Evaluation** | | | **Final CMOC** |
|  |  | **Phase I** | **Phase II** | **Phase III** |  |
| When primary care providers do not regard paramedics as diagnosticians (O) they employ them in assessment-only roles (C) until they develop trust in the paramedic’s capabilities to function effectively in primary care (M) | (Abrams et al., 2020; K. A. Abrashkin et al., 2016; Burns, 2018; Clay & Stern, 2015; Cope, 2015; Flomenbaum, 2017; Hambleton Richmondshire and Whitby Clinical Commissioning Group, 2014; Huang et al., 2018; Moule et al., 2018; NHS Salford Clinical Commissioning Group, 2016; North Dakota Center For Nursing, 2014; Ruest et al., 2017; Schofield et al., 2020). |  |  | *“…some of the stuff that they come in for with the GP, the tricky stuff, I don’t think [the paramedic would] have experience*…” (UK403: Administrative Support Staff).  *“*[the paramedic] *can take some patients but then they still need to be discussed afterwards.”* (UK802: Practice Manager).  *“I can't be in two places at the same time, but they allow me to effectively by having assessed the patient on my behalf.”* (UK702: Practice Partner).  *“…when you’re talking to a physician and, if you have the proper training for a skill – you’re explaining the medical procedure – medication that you would like to give a client – and they’re confident in your skills… sky’s the limit, and they could write you a prescription for whatever you need, or support you in anyway you need for patient care*.” (CAN303: CP Supervisor) | CMOC 7: When primary care providers do not regard paramedics as diagnosticians (O) they employ them in assessment-only roles (C) until they develop trust in the paramedic’s capabilities to function effectively in primary care (M) |
| When the employer does not consider the paramedic to have the skills and competencies relevant for their needs (C) employers may be less likely to employ paramedics (O) because they do not consider them to be useful (M) | (Barr, 2011; Burns, 2018; Cameron & Carter, 2019; Imison et al., 2016; Long, 2017; Martin & O’Meara, 2019; Mason et al., 2012; Moule et al., 2018; Sawyer & Coburn, 2017; Scott & Carney, 2004; Wagstaff & Mistry, 2020). |  |  |  | CMOC 8: When the employer does not consider the paramedic to have the skills and competencies relevant for their needs (C) employers may be less likely to employ paramedics (O) because they do not consider them to be useful (M) |
| When paramedics can demonstrate that they can help to reduce the workload of GPs (C), because this is valued by GPs (M), paramedics continue to be actively recruited into primary care (O) | (Dixon, 2020; Mid Essex Clinical Commissioning Group, 2020; Mogridge, 2017; NHS Castle Point and Rochford Clinical Commissioning Group and NHS Southend Clinical Commissioning Group, 2019; Northumberland Clinical Commissioning Group, 2016; Proctor, 2019; RSM UK Group, 2017; Schofield et al., 2020; Sibley et al., 2018; Spence, 2017; Ulintz, 2017; Watkins, 2020). |  |  | *“We're glad to have them because there’s a limit to how much we ourselves and our salaried doctors are able to assess patients and some patients just need to be assessed as to, you know, have they got heart failure, have they got chest infection or is it a combination of both. So we value them”* (UK702: Practice Partner). | CMOC 9: When paramedics can demonstrate that they can help to reduce the workload of GPs (C), because this is valued by GPs (M), paramedics continue to be actively recruited into primary care (O) |
| Paramedics are actively recruited into primary care (O) when gaps within the workforce exist (C) because they are perceived to be able to support general practice (M) | (Cameron & Carter, 2019; Goldberg, 2014; Mulholland et al., 2009; Pang et al., 2019; Turner & Williams, 2018). |  |  | *“If I was looking to fill a GP slot within the partnership, I’d be looking for a GP, if I was looking for maybe a salaried GP and couldn’t get one but I could get a paramedic practitioner, then I’d quite happily take one.”* (UK1302: GP Trainer).  *“…the NHS is changing and I think we have to go with it and increase care and capacity to patients in whatever way we can. I don’t think, oh god I’m a GP, you shouldn’t be doing my work, because I think at the end of the day if we can see more patients and treat them better, I think that’s what we’re here to do really.”* (UK603: Salaried GP). | CMOC 10: Paramedics are actively recruited into primary care (O) when gaps within the workforce exist (C) because they are perceived to be able to support general practice (M) |
| When GPs are provided with allocated during their working day to provide support to paramedics in primary care roles (C), they are more likely to offer clinical supervision (O) because they are empowered to do so (M) |  |  |  | *“So I think [paramedics] supplements* [primary care] *really well as long as the support is there…”* (UK603: Salaried GP).  *“I think it’s important that there’s actually some time allocated in the working week for the paramedic to actually discuss cases that they found difficult or challenging, with an experienced GP.”* (UK502: Salaried GP). | CMOC 11: When GPs are provided with allocated during their working day to provide support to paramedics in primary care roles (C), they are more likely to offer clinical supervision (O) because they are empowered to do so (M) |
| GPs believe (O) that paramedics with substantial experience in the ambulance service (C) are better prepared to make a successful transition and work effectively in the primary care workforce (M) |  | This idea of substantive experience was found in job descriptions included in the realist review, where an essential criterion commonly outlined the need for five years of experience as a paramedic (Eaton et al., 2021). In phase I of this research (Chapter 4), most respondents had 3–5 years (n=93) or 6–10 years (n=93) experience of working as a paramedic prior to working in primary care. |  | *“…[experience] in emergency medicine is more helpful for, you know, to work as a GP… and I'm sure the ambulance service is the same. You know you get that, you know you learn so much. You just encounter so many different problems in a short space of time.”* (UK203: Practice Partner)  *“a paramedic who's been working for a good 5/10 years you know on the ambulances so has had lots of experience.”* (UK304: Practice Partner) | CMOC 12: Paramedics with substantial experience in the ambulance service (C) are perceived to be better prepared by GPs (M) to make a successful transition into the primary care workforce (O) |

| **Paramedic Perspectives** | | | | | |
| --- | --- | --- | --- | --- | --- |
| **Initial CMOC** | **Realist Review** | **Realist Evaluation** | | | **Final CMOC** |
|  |  | **Phase I** | **Phase II** | **Phase III** |  |
| Paramedics who perceive their role as a generalist (C), will look for opportunities for employment in primary care (O) because they believe they can enjoy and work in that environment (M) | (Booker & Voss, 2019; Brown, 2017; Clarke, 2018; Daly, 2012; Eaton et al., 2018; Health Education England, 2018b; Nolan, 2013) | “*Paramedics are very competent primary care practitioners due to their generalist backgrounds*” (RID 11; Advanced Paramedic) | *“…it's the origins of paramedics that enable them to work in an advanced practice capacity* [in primary care] *and be effective at it.”* (Immersion Journal) |  | CMOC 13: Paramedics who perceive their role as a generalist (C), will look for opportunities for employment in primary care (O) because they believe they can enjoy and work in that environment (M) |
| Paramedics working in rotational roles between emergency medical services and primary care (C) feel able to apply knowledge learnt in one area to another (M) to provide improved patient care (O) |  | *“My rotational role increases understanding between ambulance & primary care, increases my knowledge & understanding…*” (RID 36; Specialist Paramedic). |  | *“I think working in* [the ambulance service] *compliments primary care and I think primary care really does compliment working for* [the ambulance service]*.”* (UK1401: Advanced Paramedic Practitioner)  *“I feel like doing community paramedic has improved my frontline, because now I ask different questions. My questions aren't only geared to the triaging life and death situations, I'm trying to formulate a diagnosis… go to the more whole health.”* (CAN201: Community Paramedic) | CMOC 14: Paramedics working in rotational roles between emergency medical services and primary care (C) feel able to apply knowledge learnt in one area to another (M) to provide improved patient care (O) |
| Paramedics who are comfortable with their professional identity (C) experience less work-related frustration and stress in primary care (O) because they understand their position within the workforce (M) |  |  | An “understanding [of] professional identity and role within the team” (Immersion Journal) was considered to be an important component for paramedics to enter primary care work, especially since “paramedics are not giving up their identity and substituting for other healthcare staff” (Immersion Journal). | “*A lot of my patients ask me the question, oh so what are you now? Or are you still a paramedic? And I think my response to them is always yeah, I’m still a paramedic. No, I don’t feel like a typical paramedic in the fact that I don’t work on an ambulance and I don’t wear green and I don’t pick people up off the floor and do those things that I think everybody thinks of as a paramedic, I don’t go to* [road traffic collisions]*, I don’t do stuff like that, but I’ve still got that knowledge and I’ve still had that training and yeah, I feel like a paramedic but I feel like I’m a paramedic who just works in a different setting.*” (UK601: Paramedic Practitioner) | CMOC 15: Paramedics who are comfortable with their professional identity (C) experience less work-related frustration and stress in primary care (O) because they understand their position within the workforce (M) |
| Paramedics working in primary care perceive colleagues as lacking a comprehensive grasp of their duties, or those who do not conform to the majority's interpretation of the role (C) they tend to "other" those individuals (O) because they are protective toward their profession (M) |  |  | This CMOC encapsulates the discussions observed during the analytic auto-netnography, (Chapter 5).  This shows that individuals outside the paramedic circle in primary care, including paramedics in the ambulance service, clinicians, non-clinical primary care staff, and policymakers, were seen as outsiders in understanding the role. This relates to the paramedics' identity shaped by their unique work environment, fostering protectiveness of their role when among fellow primary care paramedics. | *“I am still a paramedic but I don’t think I’m doing the job that is traditionally seen as paramedicine”* (UK1301: Advanced Paramedic Practitioner) | CMOC 16: Paramedics working in primary care perceive colleagues as lacking a comprehensive grasp of their duties, or those who do not conform to the majority's interpretation of the role (C). They tend to "other" those individuals (O) because they are protective toward their profession (M) |
| Fear of loss of personal professional registration (M) remains important for paramedics in primary care (C) who consider a heightened level of professional responsibility in comparison to their roles in the ambulance service (O) |  |  |  | Paramedics who were interviewed recognised their accountability in primary care, and considered it to be heightened in regards to their previous work in the ambulance service (as in Chapter 6). This accountability was developed “*from the ambulance service [where you] are drummed into you to cover your backside and protect your registration*” (UK101: Paramedic Practitioner). | CMOC 17: Fear of loss of personal professional registration (M) remains important for paramedics in primary care (C) who consider a heightened level of professional responsibility in comparison to their roles in the ambulance service (O) |

| **Perceived contribution to primary care teams** | | | | | |
| --- | --- | --- | --- | --- | --- |
| **Initial CMOC** | **Realist Review** | **Realist Evaluation** | | | **Final CMOC** |
|  |  | **Phase I** | **Phase II** | **Phase III** |  |
| When emergency medical services use rotational models for paramedics to work in both the ambulance service and primary care (C) the needs of both organisations are more likely to be met (such as EMS workforce retention and increased workforce capacity in primary care) (O) because paramedic staff retention rates and satisfaction is higher (M). | (Eaton, 2017; Goldberg, 2014; Health Education England, 2018c; B. Jones, 2020; London Ambulance Service, 2016; Mason et al., 2007a; Misner, 2005; NHS Leeds Clinical Commissioning Group, 2018; Oxford Primary Care Commissioning Committee, 2019; Primary Care One, 2020; Turner & Williams, 2018; Wickware, 2018) | *“I find that having both* [ambulance work] *and* [primary care] *works well in terms of gaining a better understanding for my patient management.”* (RID 17; Paramedic). | *A short thread on Twitter today, about the use of clinical skills in one setting being transferred and used in another. This is in the context that paramedics are expected to transfer skills from the ambulance service into primary care, but this does not work for paramedics in primary care to bring their skills into the ambulance service if they work ‘bank’ on a zero-hour contract. The example used is the ability to undertake urinalysis and liaise with a GP for relevant treatment – avoiding attendance at hospital and GP practice. However, as this is outside the scope of role for ambulance paramedics, it is not supported by my Trusts – leading to frustration, and threats that paramedics will discontinue their bank work.* (Immersion Journal). | *“…we had an informal agreement that* [primary care providers] *wouldn’t poach any of our staff, but everybody said that they got an offer – ‘Would you come and work for us full-time?’ – and nobody left. That was really interesting, so we retained everybody on a rotational model…”* (UK1101: Advanced Paramedic Practitioner).  *“So the ability to flip back and forth* [between EMS and primary care] *maybe be good for someone working that position, so they don’t ever feel like they're giving up something. So if they have an autonomy to move back and forth, it would probably help longevity and stuff like that*.” (CN201: Community Paramedic).  “…he’s six weeks on and six weeks off, so we can’t depend on his presence, so he’s always on top of what we’re doing, what we already provide, so to know that he’s here… for example, today he’s taken two visits off me, and.. what that’s meant is probably a good hour of my time… That’s what it means when he takes home visits off us – it’s physical time.” (UK803: Salaried GP). | CMOC 18: When the use of emergency medical services rotational models for paramedics enables them to work and transfer their skills between the ambulance service and primary care (C) the workforce needs of both organisations are more likely to be met (such as EMS workforce retention and increased workforce capacity in primary care) (O) because paramedic staff satisfaction is higher (M). |
| Paramedics are considered for recruitment into the primary care workforce (O) when employers perceive them as an addition to the team (M) due to the availability of adequate financial reimbursement for their roles (C) | (Baird et al., 2020; NHS England, 2021; Schofield et al., 2020). |  |  | “*I think ARRS for us was probably the reason why we actually plumped for a paramedic*” (UK102: Practice Partner) | CMOC 19: Paramedics are considered for recruitment into the primary care workforce in England (O) when employers perceive them as an addition to the team (M) due to the availability of adequate financial reimbursement for their roles (C). |
| When the existing skills and knowledge of paramedics are perceived by commissioners and stakeholders to correlate well into primary care (C) paramedics are actively recruited into primary care (O) because what they can offer is valued (M) | (Clarke, 2018; Fisher et al., 2019; Health Education England, 2017, 2018a, 2018b, 2021; Health Education England & College of Paramedics, 2016; NHS England, 2015; NHS Wales, 2018; Oxford Primary Care Commissioning Committee, 2019; Wessex Academic Health Science Network, 2017) |  |  | *“…we then started to carve out our own role in managing those patients in what we perceived as our practice environment, being in the community”* (S102: Operations Lead). | CMOC 20: When the existing skills and knowledge of paramedics are perceived by commissioners and stakeholders to correlate well into primary care (C) paramedics are actively recruited into primary care (O) because what they can offer is considered useful (M) |
| Paramedics who are judged by employers to lack clinical experience and education (C) are employed in an eyes and ears approach in primary care (O) because they lack the clinical acumen sufficient for primary care (M) |  | Data gained from phase I of the realist evaluation demonstrated that when paramedics have a lack of clinical experience or knowledge, their ability to develop sufficient clinical acumen to work in primary care is decreased and they are subsequently employed in an assessment-only (‘eyes and ears’) role. This was demonstrated through quantitative analysis (Appendix J, Appendix K).  *“work under the supervision of a GP trainer”* (RID 287: Specialist Paramedic)  *“intensity of supervision has reduced with time and confidence/capability, but have the opportunity to discuss cases or get second opinion at any time”* (RID 135: Advanced Clinical Practitioner). |  |  | CMOC 21: Paramedics with limited clinical experience and education (C) are usually employed in home visiting roles within primary care (O) because their employers judge they have insufficient clinical expertise for more challenging clinical decisions (M) |
| When paramedics are prevented by legislation or policy from seeing the full range of conditions that present to primary care (C), they feel frustrated in their role (O), because they feel they cannot contribute in the fullest way (M) |  | The impact of legislation or policy restricting paramedic clinical practice was found in phase I, where the inability to prescribe Schedule 2 controlled medicines (outlined in The Misuse of Drugs Regulations 2001), or to provide patients with a Statement of Fitness for Work were frequently cited as frustrations for paramedics. | Supported from the information gathered during phase II, where prescribing – particularly of controlled medicines – was a frequent topic of concern across the social media channels. Data from phase II confirms this is about patient care, and avoiding duplication in appointments or impact on GPs, holding true to the mechanism proposed in the CMOC. |  | CMOC 22: When paramedics are prevented by legislation or policy from seeing the full range of conditions that present to primary care (C), they feel frustrated in their role (O), because they feel they cannot contribute in the fullest way (M) |
| When paramedics are prevented by legislation or policy from seeing the full range of conditions that present to primary care (C), other clinicians in the primary care workforce are frustrated (O), because this increases their workload (M) |  |  |  | “*It’s strange what they are and aren’t allowed to prescribe, it’s odd signing scripts for co-codamol when potentially they don’t need to sign scripts for what I would consider bigger, harder drugs, I find that a little bit odd*” (UK1302: GP Trainer).  *“It’s certain medications – obviously because , if he wants CDs, (paramedic’s name)…he can’t prescribe controlled drugs – so that has to be with the GP.”* (UK902: Practice Manager).  “*we’re doing that on top of our day-to-day work. It’s very hard at the moment – we can’t recruit GPs so, fortunately, none of us ever make them feel that they can’t ask questions, but it’s hard for us – I can’t lie*.” (UK1002: GP Trainer). | CMOC 23: When paramedics are prevented by legislation or policy from seeing the full range of conditions that present to primary care (C), other clinicians in the primary care workforce are frustrated (O), because this increases their workload (M) |
| When paramedics provide access to healthcare which otherwise would not be available (C) they are considered a community asset (O) because what they do is highly valued by patients and service commissioners and providers (M) | (Bennett et al., 2018; Blacker et al., 2009; Eaton, 2017; Hauswald et al., 2005; Martin et al., 2016; Mason et al., 2007b; Misner, 2005; Mulholland et al., 2009; O’Meara et al., 2012; Patterson et al., 2016; Pennel et al., 2016; Stirling et al., 2007; Wilcox, 2016; Wiley K., 2011) |  |  | *“the local hospital is twenty-five miles away – a fifty-minute journey by ambulance due to the winding roads away from the coast. Health access is poor in this part of the country, and the local population is heavily reliant on the services provided by the practice. Hence, he will suture, managing minor injury and perform minor operations. He’s always on standby too, he nods to the* [emergency response] *bags in the corner of the room.”* (UK 12: Fieldnotes).  *“…the good thing about community paramedics is you can formulate it to whatever your area needs. If you're in a large urban centre and it has tons of services, and your community paramedic role is going to be a lot more focused. Whereas here in county we have limited amount of resources, our community paramedic programme is very broad, because we’ll try to help wherever we can and fill gaps or be partners in patient care*.” (CN201: Community Paramedic). | CMOC 24: When paramedics provide access to healthcare which otherwise would not be available (C) they are considered a community asset (O) because what they do is highly valued by patients and service commissioners and providers (M) |

| **Concept 2: Transition to primary care roles** | | | | | |
| --- | --- | --- | --- | --- | --- |
| **Education** | | | | | |
| **Initial CMOC** | **Realist Review** | **Realist Evaluation** | | | **Final CMOC** |
|  |  | **Phase I** | **Phase II** | **Phase III** |  |
| When employers provide clinical supervision (C) and access to formal education to paramedics (C) they are better able to transition into primary care roles (O) because they feel supported (M) |  |  |  | “*I think clinical supervision, access to courses and the time to be given to actually improve skills and improve learning would be the best way to support my role at the moment.*” (UK401: Paramedic)  *“I think that needs to be structured, definitely and having spoken to the other paramedics started the same time as me, they would be like, so overwhelmed as well. I didn’t have a clue and we did pick it up, but I feel like it would have been so much less overwhelming if we’d have just had that structure.”* (UK501: Paramedic) | CMOC 25: When employers provide clinical supervision (C) and access to formal education to paramedics (C) they are better able to transition into primary care roles (O) because they feel supported (M) |
| Paramedics are able to transition into advanced practice roles (O) when they are supported by their employers in primary care (M) with clinical supervision (C) and access to formal education (C) | (Bowles et al., 2017; Eaton et al., 2021; Graham, 2018; Health Education England, 2021; Health Education Kent Surrey and Sussex, 2014; O’Meara et al., 2014) | *“I am at a very early stage in my 3-year ACP program (~8 months in), and therefore supervision is key to my practice as I develop my own competency and scope of practice. My primary care surgery are very supportive and helpful, and appear invested in my development.”* (RID 305: Trainee Advanced Clinical Practitioner) |  |  |  |
| As paramedics transition from EMS to primary care roles (C), they move away from their traditional scope of practice (O) because of the change in clinical conditions they have to manage (M) | (Agarwal et al., 2017; Bowles et al., 2017; Chan et al., 2019; Colver et al., 2016; Dixon, 2020; Evans et al., 2014; Griffin, 2015; Kusel & Savino, 2016; Lau et al., 2018; Long, 2017; Montera, 2016; Murray, 2018; O’Hara et al., 2012; O’Keeffe et al., 2011; Ruest et al., 2017; Stirling et al., 2007; Wiley K., 2011; Woollard, 2006) | Understanding of the clinical presentations and scope of role were developed from phase I of the realist evaluation through quantitative analysis (Appendix J). These highlight a significant move from the scope of role required of paramedics in ambulance services, both in terms of clinical examination skills, but also in terms of capabilities required for primary care (Appendix I). | Supported in phase II, where conversations between paramedics revealed that “actually the scope of role being practised is actually far wider than even that set out in the Roadmap.” (Immersion Journal). | expansion in scope of role was also observed during phase III, where paramedics were witnessed to deal with dermatology and medication management for chronic conditions – two distinct areas lacking in the traditional scope of role for paramedics in UK ambulance services (Joint Royal Colleges Ambulance Liaison Committee, 2017).  An expansion in the scope of role was also outlined by community paramedics in Canada, who are able to undertake point of care blood and microbiology testing from a procedural skill point of view and now undertook these skills more regularly than their emergency care skills (CN1: Fieldnotes). | CMOC 26: As paramedics transition from EMS to primary care roles (C), they move away from their traditional scope of practice (O) because of the change in clinical conditions they have to manage (M) |
| Due to the geographical nature of primary care work (C), paramedics tend to use online social spaces to exchange insights regarding their role (O) and support each other (O) because it is a convenient way to engage with others (M) |  |  | *“I’m reminded of the importance of this community for sharing resources and building learning. But, for paramedics in primary care not on Facebook, what communities of practice do they belong to?“* (Immersion Journal). | *“It’s double-edged – it’s wonderful because I’m left alone – do what I do – lots of autonomy but, at the same time, there isn’t a community practice – there isn’t somebody to kick things round with…”* (CN301: Community Paramedic).  *“You always had that buffer. You had somebody else there going, yeah, that’s right or no, that’s wrong. In primary care, you’re on your own… solitary probably is a better word…”* (UK1301: Advanced Paramedic Practitioner).  *The other thing for me, moving out into primary care, when you say about barriers, it’s quite lonely because there is nobody else that’s done it before, so you’re well away from the Ambulance Service, a system that you know, you recognise and you’ve been through...”* (UK1101: Advanced Paramedic Practitioner). | CMOC 27: When professional isolation exists for paramedics in primary care (C), paramedics tend to use online social spaces to exchange insights regarding their role (O) and support each other (O) because it is a convenient way to engage with others (M) |
| The lack of a structured curriculum to support primary care education (C) means that paramedics learn through practical experience of working in primary care (M) leading to variations in the implementation of the role (O) |  | *“Someone on Facebook has requested recommendations for courses to improve their examination and assessment of musculoskeletal conditions. Replies range from e-learning for health, local conferences/study days in some PCNs, to diplomas organised by independent companies. I guess this is the problem with no standardised education level or content for working in primary care.”* (Immersion Journal). |  | “*I would say is that academia is one thing, experience is another and the thing that will make a clinician brilliant is the experience*.” (UK1201: Advanced Paramedic).  *“I haven’t particularly done any courses – I’ve done a Blood Interpretation course – that’s probably the only course I’ve done, but it’s just general day-to-day, you pick things up and you go, ‘Oh, I didn’t know that – I didn’t know what the meant!’ but I haven’t got a list of what I have learnt – just general knowledge.”* (UK701: Home visiting paramedic).  *“I know how to find what I need to know about chronic disease. But we don’t necessarily get taught what we need to know. So, I learnt how to go about finding it”* (CN301: Community Paramedic). | CMOC 28: Lack of a structured curriculum to support primary care education (C) means that paramedics learn through practical experience of working in primary care (M) leading to diverse approaches to role implementation (O) |
| When paramedics recognise that working in primary care requires additional study (C) they will often undertake this in their own time (which has an impact on their work/life balance) (O) because they want to do their job well (M) |  |  |  | *“I will still spend one of my days off just studying because I just want to have a bit more ease with knowing stuff easier with patients.”* (UK1301: Advanced Paramedic Practitioner).  *“…so I spend one afternoon at home a week doing e-learning…”* (UK801: Urgent Care Practitioner Paramedic).  *“It was only one day a week at uni, and then I did study in the evenings, but when you sign up to these things, you know you’re gonna have to put a bit of your own time into it, but it worked quite well.”* (UK1001: Paramedic Practitioner)  *“I do lots of professional development – knowledge acquisition. I’ll see something that I’m not familiar with – I will be looking at that this weekend. You know what I mean? – just because I need to know more. You know like that kind of stuff – so, I’ll do that, for sure – all the time – but I think that’s just professional development.”* (CN301: Community Paramedic). | CMOC 29: When paramedics recognise that working in primary care requires additional study (C) they will often undertake this in their own time (which has an impact on their work/life balance) (O) because they want to do their job well (M) |

| **Supervision** | | | | | |
| --- | --- | --- | --- | --- | --- |
| **Initial CMOC** | **Realist Review** | **Realist Evaluation** | | | **Final CMOC** |
|  |  | **Phase I** | **Phase II** | **Phase III** |  |
| When paramedics are clinically supported in general practice (C), because they feel better supported (M) they will continue to advance and develop their capabilities and confidence within their role (O) | (Baird et al., 2020; Brooke Petter Associates, 2019; Chellappa et al., 2018; Hauswald et al., 2005; Health Education Kent Surrey and Sussex, 2014; Moule et al., 2018; Ruston & Tavabie, 2011) | *“Intensity of supervision has reduced with time and confidence/ capability, but have the opportunity to discuss cases or get second opinion at any time.”* (RID 135: Advanced Clinical Practitioner) | *“A common feature has been those struggling with placement or supported hours due to the lack of supervision available during Covid-19. This has had a significant effect on ability to complete modules – but more fundamentally to have the relevant supported exposure to case presentations in primary care.”* (Immersion Journal) | *“…the senior partner here – before he retired – he said to me, ‘Whatever we threw at ya, you took on and you learnt from it.’ He said, ‘When you first started here, you were a paramedic’ – he said, ‘You’re more than that now,’ really. He says, ‘Your skill level’s come on because you’ve developed and you took on the feedback and developed”.* (UK901: Paramedic Practitioner) | CMOC 30: When paramedics are provided with clinical feedback and supervision in general practice (C), because they feel supported and valued (M) they will develop their capabilities and confidence within their role (O) |
| When paramedics are provided with clinical feedback, advice, and supervision in general practice (C), because they feel better supported (M), they have higher satisfaction with their role (O) |  | *“I have a GP trainer as my mentor who is a very experienced GP. The knowledge that I gain from spending time with her is valuable to me as it helps me improve as a clinician and means I’m very happy in my work.”* (RID 240: Emergency Care Practitioner) | *“…several comments from primary care paramedics – outlining the cancellation of teaching/clinical supervision this year as their practices have responded to the Covid-19 pandemic. One went as far to say that they had left primary care precisely because of this…”* (Immersion Journal) | *“I think clinical supervision, access to courses and the time to be given to actually improve skills and improve learning would be the best way to improve my role at the moment.”* (UK401: Paramedic) |  |
| When clinical supervision is accessible for paramedics (C), they have higher satisfaction with their role (O) because they feel better supported (M) |  | *“I have access to GP support when required and regularly discuss more complex patients for learning and development*” (RID 203: Consultant Paramedic)  85.6% indicated that they received clinical supervision in their role, however only 8% reported that they experienced a regular and structured clinical supervision model that adequately met their needs. |  | “*If I didn’t have that support there or I didn’t feel I could approach the doctors, that would be a whole different ball game because I would feel anxious going to ask, but it doesn’t bother me ‘cause I know I’ve got that support and guidance and I wouldn’t just guess.”* (UK501: Paramedic) |  |

| **Experience** | | | | | |
| --- | --- | --- | --- | --- | --- |
| **Initial CMOC** | **Realist Review** | **Realist Evaluation** | | | **Final CMOC** |
|  |  | **Phase I** | **Phase II** | **Phase III** |  |
| Paramedics at a junctional point within their career (C) value the opportunity to develop themselves (M) and so look for opportunities for employment in primary care (O) | (Brown, 2017; NHS Wiltshire, 2020) | “*To develop my career because unable to develop in the ambulance service*.” (RID 46: Emergency Care Practitioner)  “*Career Progression via the paramedic pathway to primary care. To further develop my clinical skills and knowledge. To have the opportunity to undertake an ACP Msc and independent prescribing*” (RID 270: Primary Care Practitioner) |  |  | CMOC 31: Paramedics at a junctional point within their career (C) value the opportunity to develop themselves (M) and so look for opportunities for employment in primary care (O) |
| Paramedics are pluripotent (C). Because of the breadth of issues with which they can deal (M) paramedics are considered a useful addition to the primary care team (O) | (Brackenridge, 2018; Eaton, 2017; Kizer, 2016; NHS England, 2016; Wilcox, 2016) |  |  | *“…it is absolutely great that we are transitioning into this space. It’s exactly where we need to be and we’re definitely capable of doing it. I think it helps relieve pressures by bringing another AHP into a system” (UK1101: Advanced Paramedic Practitioner)*  *“*[paramedics] *do a lot of different types of patients rather than physiotherapists who tend to be much more linked to specific people who need it, musculoskeletal problems. So they can be more diverse in the types of patients that they can see.”* (UK102: GP Partner) | CMOC 32: Paramedics are pluripotent (C). Because of the breadth of issues with which they can deal (M) paramedics are considered a useful addition to the primary care team (O) |
| Paramedics who are dissatisfied with their work in the ambulance service (C) will look for opportunities for employment in primary care (O) because they believe this will afford them a better work/life balance(M) |  | “*To get away from shift work, have a better work/life balance and thought primary care was the way forwards after being a paramedic on the road for a long time. Didn’t enjoy the trauma aspect either on the road anymore.”* (RID 107: Paramedic) | “*Paramedics are expected to provide definitive care through the ambulance service due to a high degree of low acuity/primary care prescriptions – so no wonder paramedics look for opportunities to move to primary care (and not work nights”* (Immersion Journal) | *“…I was struggling after a couple of horrendous jobs I went to, so I went to two cardiac arrests in quick succession, and really affected my mental health… and I just needed to prioritise my own wellbeing to make a move out of the Ambulance Service.”* (UK701: Home Visiting Paramedic)  *“When I first joined the ambulance service, you might have four or five jobs in a 12 hour shift and some night shifts you wouldn't go out at all. Where now I think you book on your 12 hours and you are out 12 hours, you've got a fight to get a break, you're always late from your 12 hour shift…Where now there's a lot more opportunities, especially in primary care. You know come and work with us, work Monday to Friday, have a normal life, have a weekend off, have leave when you want it you know.”* (UK301: Specialist Paramedic) | CMOC 33: Paramedics who are dissatisfied with their work in the ambulance service (C) will look for opportunities for employment in primary care (O) because they believe this will afford them a better work/life balance(M) |
| Paramedics who are experienced by virtue of their work in the ambulance service (C) fulfil an increased clinical role in primary care (O) because they have practical experience to draw upon (M) |  | “*The Paramedic ACP provides a skilled independent clinician who can manage a large variety of presentations autonomously in environments that other clinicians are less comfortable working in.”* (RID 26: Trainee Advanced Clinical Practitioner)  “*We are used to seeing acute patients, often needing to use problem solving skills having come from an ambulance background.”* (RID 28: Paramedic Practitioner)  *“I feel I am a versatile and flexible clinician and my practice can use me and my skills in various capacities.”* (RID 117: Emergency Care Practitioner) | “*A short thread on Twitter today, about the use of clinical skills in one setting being transferred and used in another”* (Immersion Journal)  *“The value paramedics bring to primary care is built on 999 experience – significant experience in spotting sick people makes a safer clinician in primary care.”* (Immersion Journal) | *“With the Ambulance Service, you just see everybody, everything, don’t you, that calls an ambulance, you will see”* (UK1001: Paramedic Practitioner)  “*I’ve got a couple of seasons under my belt and I’ve got quite a lot of pre-hospital experience with the ambulance service. Before I undertook my master’s I’d been a paramedic for over ten years, so I had a solid grounding of patient assessment skills that you do pick up over the years, and not only that, also your continual development and your learning of other conditions and how conditions are managed.”* (Uk1401: Advanced Paramedic Practitioner) | CMOC 34: Paramedics who are experienced by virtue of their work in the ambulance service (C) fulfil an increased clinical role in primary care (O) because they have practical experience to draw upon (M) |
| When paramedics are not allowed to see some patient groups due to the clinical guidelines that exist in their work in ambulance services (C) they are prevented from gaining experience in the whole range of conditions that present to primary care (O) because they don't have these relevant opportunities to learn from (M) |  | Patient groups not seen reported to be:   - Chronic conditions - Men’s health - Palliative Care - Children <2 years - Women’s health |  |  | CMOC 35: When paramedics are not allowed to see some patient groups due to the clinical guidelines that exist in their work in ambulance services (C) they are prevented from gaining experience in the whole range of conditions that present to primary care (O) because they don't have these relevant opportunities to learn from (M) |
| When paramedics have newly transitioned into working in primary care settings (C) effective time management poses a challenge for them (O) because they lack the experience needed (M) |  |  | “…for home visits between sessions, I had 2 hours and would see up to 4 patients. I didn’t need to report back to a GP until at the end of the day, which is when my catch-up time was. The longer I was there, the more visits I needed to do, in less time.” (Immersion Journal)  *“Condensing any patient assessment and history take into a 10-15 appointment was considered to be the biggest learning curve when moving into primary care. I agree, we just don’t have the time pressure in the ambulance service.”* (Immersion Journal) | *“…you could be with a patient for an hour in the ambulance service…but that’s not Primary Care – that’s one of the obstacles I had to overcome. I still have 15 minutes – GPs only have 10 minutes – I still have 15-minutes appointments – but it was learning how to deal with the patients and sometimes you run a little bit over – sometimes you don’t.”* (UK901: Paramedic Practitioner)  *“I’m still struggling to keep on time and that’s me a couple of years into the job whereas at the beginning, it was near impossible.”* (UK1301: Advanced Paramedic Practitioner) | CMOC 36: When paramedics have newly transitioned into working in primary care settings (C) effective time management poses a challenge for them (O) because they lack the experience needed (M) |
| When paramedics have no previous experience of working in primary care (C), they are surprised about the magnitude and type of work they are required to do (O) owing to an absence of adequate preparation for such responsibilities (M) |  |  | *“However, whilst busy, the ambulance service is nowhere near as busy as primary care.”* (Immersion Journal)  *“…pieces of advice include:*  *• Being prepared for the increase of workload.*  *• That it is rewarding, but hard work.”* (Immersion Journal) | *“it was a really, really, really steep learning curve*” (UK101: Paramedic Practitioner)  “*Moving into primary care, there’s always that first little imposter syndrome. Yes I know what I can do with emergency care. Moving to primary care, it’s a big step and a steep learning curve.”* (UK401: Paramedic) | CMOC 37: When paramedics have no previous experience of working in primary care (C), they are surprised about the magnitude and type of work they are required to do (O) owing to an absence of adequate preparation for such responsibilities (M) |
| Paramedics consider that their experiences in the ambulance service (C) are important in preparing them to work effectively in primary care (O) as working in the emergency ambulance setting has enabled them to develop their professional praxis (M) |  |  |  | “*I would say that you would need more experience on the road to come in because you still need to kind of have that sixth sense and understand when someone's not quite right, maybe their observations all look alright but actually there's something about them that's not recognising unwell patients because I think that's probably what we're quite good at.”* (Uk101: Paramedic Practitioner)  “*Our skill and our background and our training as autonomous clinicians is that we get dropped into any situation and we have to figure it out and work with the resources that we’ve got, and that is what makes us dynamic when you move into primary care.”* (UK1101: Advanced Paramedic Practitioner) | CMOC 38: Paramedics consider that their experiences in the ambulance service (C) are important in preparing them to work effectively in primary care (O) as working in the emergency ambulance setting has enabled them to develop their professional praxis (M) |
| Experience paramedics in primary care (C) complement the role of the GP (M) and are able to increase workforce capacity (O) by improving patient access to consultations (O) |  | “*We compliment the PC team. We can bridge between GP type work as well as the more acute presentations that we may be more used to dealing with*.” (RID 32: Paramedic) |  | “These people or some of them haven't been to a doc in six years… when you're in someone’s home dealing with their health concerns, keeping them at home, keeping them out of hospital, keeping them as healthy as we can keep them, safe at home as we can” (CN101: Community Paramedic)  “*Without a doubt, paramedics have the ability to plug a workforce gap, I think but more than that, that's disingenuous to say that’s all they can do. I'm saying they can plug a workforce gap, they can be a completely valuable member of your team”* (UK102: GP Partner)  *“The workload, that’s off the GPs, off the nurses. I’ve got two home visits sat there, that’s two home visits that GP doesn’t have to do.”* (UK801: Urgent Care Practitioner) | CMOC 39: Experience paramedics in primary care (C) complement the role of the GP (M) and are able to increase workforce capacity (O) by improving patient access to consultations (O) |

| **Concept 3: Roles and responsibilities** | | | | | |
| --- | --- | --- | --- | --- | --- |
| **Working in a team** | | | | | |
| **Initial CMOC** | **Realist Review** | **Realist Evaluation** | | | **Final CMOC** |
|  |  | **Phase I** | **Phase II** | **Phase III** |  |
| When the professional role boundaries of paramedics overlap with existing health care professionals in General Practice (C) there may be resistance of the paramedic role and responsibilities by these other health care professionals (O), because they feel threatened (M) | (Barr, 2011; Coleman et al., 2011; Imison et al., 2016; Rasku et al., 2019; Thurman et al., 2020) | “*not feeling part if the primary care team*” (RID 104: Specialist Paramedic)  *“still feel that there is a lack of understanding, or clear delineation between roles and this can mean I’m not welcomed*” (RID 305: Trainee Advanced Paramedic Practitioner) |  |  | CMOC 40: When the professional role boundaries of paramedics overlap with existing health care professionals in General Practice (C) there may be resistance of the paramedic role and responsibilities by these other health care professionals (O), because they feel threatened (M) |
| Paramedics experience frustrations in their role (O) when their role and responsibilities are unclear (C). When this occurs, paramedics are less likely to be empowered (M) | (M. S. Leyenaar, McLeod, et al., 2019; M. S. Leyenaar, Strum, et al., 2019; Long, 2017; Montera, 2016; Murray, 2018) | “*That on occasion other clinicians think I can do more than I’m comfortable with doing”* (RID 57: Emergency Care Practitioner)  *“…the scope of role so broad its hard to delineate and develop formally.”* (RID 121: Paramedic) |  |  | CMOC 41: Paramedics experience frustrations in their role (O) when their role and responsibilities are unclear (C). When this occurs, paramedics are less likely to be empowered (M) |
| When the paramedics capabilities have been demonstrated (C) they are viewed by practice staff as a credible addition to the team (M) and are accepted into the practice workforce (O) | (Burns, 2018; Flomenbaum, 2017; Kizer, 2016; Long, 2017; Martin & O’Meara, 2019; Moule et al., 2018; O’Meara et al., 2012; Schofield et al., 2020; Thurman et al., 2020; Turner & Williams, 2018) |  |  | *“…he’s a good support to the nursing team, so he can do basically anything, ((Name)) can – he’s fabulous”* (UK1002: Practice Manager)  *“it's actually turned out that they have taught me so much and vice versa*.” (UK703: Home Visiting District Nurse) | CMOC 42: When the paramedics capabilities have been demonstrated (C) they are viewed by practice staff as a credible addition to the team (M) and are accepted into the practice workforce (O) |
| When the role of paramedics is clear and well defined (C) the paramedic integrates well in the primary care workforce (O), because their contribution is clear (M) | (Flomenbaum, 2017; Kizer, 2016; M. Leyenaar et al., 2018; M. S. Leyenaar, Strum, et al., 2019; Long, 2017; Martin & O’Meara, 2019; O’Meara et al., 2012; Thurman et al., 2020; Turner & Williams, 2018) |  | *“A small (happy) thread on Twitter – talking about the successes of PCNs. With general responses regarding vaccine delivery (in the minds of many recently, I guess) also outlined is the extra resources afforded to practices under the ARRs scheme – particularly paramedics, who are versatile and flexible, both in home visiting and routine appointments.”* [Immersion Journal] | “*We all work together really well and pass things around to the most appropriate people to deal. Yeah, very integrated, I would say.”* (UK1001: Paramedic Practitioner) | CMOC 43: When the role of paramedics is clear and well defined (C) the paramedic integrates well in the primary care workforce (O), because their contribution is clear (M) |
| Working in primary care (C) requires paramedics to reframe their professional identity (M) in order to integrate into the workforce (O) |  |  | *“…paramedics are not giving up their identity and substituting for other healthcare staff – it's the origins of paramedics that enable them to work in an advanced practice capacity and be effective at it.”* [Immersion Journal]  *“So, what do I think are the bare essentials for paramedics to work in primary care?*  *… Understanding professional identity and role within the team”* [Immersion Journal] | *“It does feel like a different role altogether, from being a paramedic. When I was with the Ambulance Service”* (UK1001: Paramedic Practitioner)  *“I think by trade, I’m still a paramedic but in what people would recognise as a paramedic isn’t what I do now. I still introduce myself as a paramedic ‘cause that is what I am. It’s a loaded question. I am still a paramedic but I don’t think I’m doing the job that is traditionally seen as paramedicine* [resulting in] *a kind of loss of identity as a paramedic”* (UK1301: Advanced Paramedic Practitioner) | CMOC 44: Working in primary care (C) requires paramedics to reframe their professional identity (M) in order to integrate into the workforce (O) |
| The paramedic profession (C) is poorly understood by primary care teams (O), who associate it with the provision of emergency care in an ambulance only (M) |  |  |  | *“The first thing I think of, if I’m honest, is somebody that comes in a paramedic ambulance…”* (UK604: Advanced Nurse Practitioner)  *“It's sort of like just knowing from seeing like casualty or something”* (UK103: Receptionist)  *“My idea of the job description is a competent medical professional to provide first aid – more than that – to help a patient survive in a life-threatening situation that will include, CPR, defibrillator, things like that… they are more than welcome to join in and give a hand, but it is not really what they have been trained to do in particular, which is the response to 999 and emergency medical care.”* (UK1104: Salaried GP) | CMOC 45: The paramedic profession (C) is poorly understood by primary care teams (O), who associate it with the provision of emergency care in an ambulance only (M) |
| Primary care teams consider that length of experience in the ambulance service (C) is important in preparing paramedics to work effectively in primary care (O) because they have a broad range of practical experience to draw upon (M) |  |  |  | *“Other paramedics who would be coming straight from, say, the ambulance service would be much more limited in their ability when they first arrive*.” (UK102: GP Partner)  “*So he's had a massive exposure to a massive number of ailments from you know, really minor to major trauma so I’d feel that he can deal with anything.”* (UK304: GP Partner) | CMOC 46: Primary care teams consider that length of experience in the ambulance service (C) is important in preparing paramedics to work effectively in primary care (O) because they have a broad range of practical experience to draw upon (M) |

| **Interpersonal skills** | | | | | |
| --- | --- | --- | --- | --- | --- |
| **Initial CMOC** | **Realist Review** | **Realist Evaluation** | | | **Final CMOC** |
|  |  | **Phase I** | **Phase II** | **Phase III** |  |
| For patients, the interpersonal skills of the paramedic are one of the most important components of the consultation (C). Where paramedics display empathy, active listening and a holistic approach to the patient (M), the patient develops trust and confidence in the paramedic (O). | (Ball, 2005; Clarke, 2018; Hill et al., 2014; Lau et al., 2018; Martin & O’Meara, 2019; Rasku et al., 2019) | “*Patients feel that they are seen by a professional who listens to them”* (RID 41: Advanced Paramedic Practitioner)  “*I believe the public has a lot of trust in the paramedic profession and this impacts on patients’ confidence in you as a clinician.*” (RID 192: Emergency Care Practitioner) |  | *“the bedside manner, you know, especially when you’re feeling ill and being understanding is everything.”* (UK1102: Patient)  *“I’d just like to say he’s a real gentleman, a great paramedic, a great people’s person, he’s got great people skills”* (UK204: Patient Relative)  *“Compassion – paramedics are more compassionate, I find, than a lot of people, generally”* (CAN104: Patient) | CMOC 47: Where paramedics display empathy, active listening and a holistic approach to the patient (C) the patient develops trust and confidence in the paramedic (O) because they feel heard and respected (M) |
| Successful integration of paramedics into primary care teams (O) is attributed to the interpersonal skills and enthusiasm of the paramedic (C) because primary care team members use these to judge the type of person they work alongside (M) |  |  |  | “*I think a lot of it is personality. He gets on with everybody here in the surgery. I don’t think there’s anybody who doesn’t get on with ((name)).”* (UK1504: Practice Nurse)  “*He is a very wonderful person with a lovely character, so it’s definitely about his personality, but no – his role within the practice also.”* (UK103: Deputy Practice Manager)  *“She’s friendly. She’s open and approachable. I think they’re always the key things ultimately. I think she’s very good at engaging with others and communicating. She gets involved. She’s wanted to fit in.”* (UK502: Salaried GP) | CMOC 48: Successful integration of paramedics into primary care teams (O) is attributed to the interpersonal skills and enthusiasm of the paramedic (C) because primary care team members use these to judge the type of person they work alongside (M) |

| **Clinical role** | | | | | |
| --- | --- | --- | --- | --- | --- |
| **Initial CMOC** | **Realist Review** | **Realist Evaluation** | | | **Final CMOC** |
|  |  | **Phase I** | **Phase II** | **Phase III** |  |
| Paramedics who have a higher level of education and clinical experiences (C) fulfil an increased clinical role in primary care (O) because they have greater knowledge and capabilities (M) |  | Understanding of the clinical presentations and scope of role were developed from phase I of the realist evaluation through quantitative analysis (Appendix J). This highlights the relationship between level of education, experience as a paramedic, and the breadth of role undertaken (Appendix I). | *“The value paramedics bring to primary care is built on 999 experience – significant experience in spotting sick people makes a safer clinician in primary care.”*  *“The idea of holistic assessments has been raised again – which is linked to increased education”.*  *“Also featured today a very limited chat on education level for paramedics in primary care, outlining that Level 6 (BSc) is enough for the clinical role. I’m on the fence about this. I don’t believe Level 7 learning gives new knowledge (the facts don’t change from Level 6 to 7), but the way we think about clinical presentations in advanced practice, at Level 7, is different. Is Level 7, therefore, critical thinking and decision-making applied to patient presentations?”*  *(Immersion Journal* | *“I guess I’m going to move on a see a different class or level of patients and conditions once I’ve done the Master’s, really. I’m obviously gonna learn and I think it’s gonna be beneficial to my practice, isn’t it, to do that extra study.”* (UK1001: Paramedic Practitioner)  *“It’s not enough to have a master’s degree. You need a master’s degree plus quite a lot of experience in primary care and maybe also in your base profession”* (UK1301: Advanced Paramedic Practitioner) | CMOC 49: Paramedics who have a higher level of education and clinical experiences (C) fulfil an increased clinical role in primary care (O) because they have greater knowledge and capabilities (M) |
| Paramedics who can independently prescribe medicines (C) fulfil an increased clinical role in primary care (O) because they are able to complete a broader range of consultations (M) |  | Understanding of the clinical presentations and scope of role were developed from phase I of the realist evaluation through quantitative analysis (Appendix J). This highlights the relationship between the ability to independently prescribe and the breadth of role undertaken (Appendix I). | *“…on Twitter are comments following a commentary focused on paramedic prescribing in primary care. The other comments on the broad scope of prescribing practice paramedics require in primary care, and lists recommended resources based on practical experience of the author. Comments to this are generally positive, acknowledging the advantage of the ability for paramedics to prescribe in primary care”* (Immersion Journal). | *“*Prescribing is essential. It’s a busy environment, so you don’t want to bother GPs all the time if you can manage stuff.” *(UK1001: Paramedic Practitioner)*  *“His prescribing practice now is different to his practice when he started. He feels he can contribute more now than he could in the fifteen years in primary care before he was a prescriber”* (UK12: Field notes) | CMOC 50: Paramedics who can independently prescribe medicines (C) fulfil an increased clinical role in primary care (O) because they are able to complete a broader range of consultations (M) |
